# Remarks on Forcible Breathing and the Pneumatic Cabinet1Read before the Medical Society of the County of Erie.

**Published:** 1896-10

**Authors:** J. H. Pryor

**Affiliations:** Buffalo, N. Y. 56 Allen Street


### REMARKS ON FORCIBLE BREATHING AND THE PNEUMATIC CABINET.[1]

By J. H. PRYOR, M. D., Buffalo, N. Y.

THIS short paper has reference only to the therapeutic value of deep or forcible breathing in certain affections of the lungs. The methods employed have been to instruct the patient how to breathe deeply and slowly, accompanied by directions to take a certain number of long breaths at different times during the day, and the use of the pneumatic cabinet as an aid to secure forcible breathing. The patient is directed to remove or loosen the clothing which interferes with the free action of the thoracic and abdominal muscles, endeavor to practise abdominal breathing and take full breaths, inspiring and expiring the air slowly. At first this exercise may be limited to twenty full breaths, the first thing in the morning and the last thing at night, and the amount of exercise then gradually increased. When the patient can be out of doors it is well to recommend forcible respiration in the open air to be practised in this manner: While walking inspire slowly and count twelve. Let the expiratory effort consume as much time as the inspiratory and gradually increase the period of time by counting thirty at the same rate during each inhalation. Often patients will not follow directions unless the importance of the matter is impressed upon them. Few are accustomed to deep breathing and never breathe properly until taught how. In some cases several deep breaths are followed by dizziness, cardiac palpitation and stiffness of the chest muscles. This is especially true of women who never take any active exercise and have never filled

1. Read before the Medical Society of the County of Erie.

the lungs to the extent of their capacity. In several instances I have been unable to gain compliance on the part of a child and have been obliged to resort to the cabinet or the use of a small wooden tube, which the child will usually blow through most vigorously, because the mind is involved in the effort. Motions of the arms to assist in increased respiratory effort should be combined when advisable. Strengthening of the abdominal muscles and diaphragm may be promoted by what the athlete calls stomach pumping. The beneficial effects of forcible breathing are generally most noticeable in persons who never make the lungs perform more work than is necessary for an inactive physical existence. In certain subacute and chronic affections of the lungs and pleura, forcible breathing is one of the most valuable remedial agents in our possession. The conditions most benefited by its employment are subacute bronchitis, slowly resolving chronic pneumonia, collapsed lung after pleurisy with effusion, limited motion of the lung from pleuritic adhesions, and insufficient oxygenation in tuberculosis pulmonalis. The beneficial effects are produced by an increased supply of oxygen, promoting and equalising the circulation, mechanical distention of air vesicles, stimulating the activity of the glandular system and the flow of lymph by pressure acting as a massage of the lung and so hastening absorption of exudates.

In this climate cases of subacute bronchitis which refuse for a time to yield to medicinal treatment are common. The recovery is usually hastened by vigorous breathing. Often after twenty prolonged deep inhalations the sibilation will disappear temporarily. It is usually followed by expectoration and the disagreeable rattling is relieved. The absorbents are stirred into activity, the circulation quickened and the lungs ventilated by an increased supply of tidal air. There is no medicine so powerfully and safely stimulating as pure air in these cases. I have repeatedly noted the recovery of patients suffering from subacute bronchitis in two or three days, who secured no benefit from long-continued medicinal treatment. Inhalations of medicaments may be combined with deep inhalations, but the open air is preferable if the condition of the patient and the weather will permit. Often perspiration can be produced by deep breathing when medicines fail. When resolution is slow or stops before the exudate of croupous pneumonia has disappeared, no method of treatment is so successful as the one described. The mode of action is largely mechanical, but recovery is hastened by breathing in the open air. This is not always possible in Buffalo and the question of a change of climate must be considered. The distention of air vesicles, partially closed, by foul indoor air will not be sufficient. When necessary, especially if the patient be confined to the bed, a tin pipe or tube may be arranged to connect with the outdoor air. The patient who has suffered from pneumonia should not be pronounced well until air enters the lungs freely everywhere. Areas of consolidation frequently become the seat of tubercular infiltration. Extra play of the lung hastens absorption of the exudative deposit and distends the collapsed air vesicles. During the last five years I have had under observation several cases of so-called chronic pneumonia. They were not of the interstitial type, but of the variety characterised by continued consolidation of a part of the lung. One had recovered from acute lobar pneumonia four years previously and the others had pneumonia at times, ranging from two years to two months before coming under observation. In every instance the improvement from forcible breathing was most marked. The breath sounds changed slowly but surely from pure bronchial to vesicular or broncho-vesicular. Two of these cases still have a slightly crippled lung, but diligent practice has increased the capacity of the permeable areas and the symptoms of insufficient oxygenation have disappeared. The employment of increased pulmonary exercise to distend a portion of lung collapsed from the pressure of pleuritic effusion has long been practised as a mechanical means. As a rule, the effort is not sufficiently systematic or vigorous. When the method has been prescribed it should mean hard work, not a lazy attempt or play, and the patient should not be allowed to discontinue because coughing occurs. The cough is beneficial and particularly so when there are adhesions. Adhesions should be broken up early while they are fresh. Later they are usually only partially absorbed or stretched. In the majority of cases of collapsed or adherent lung following pleurisy, the crippled area may be made more serviceable and the compensatory work of the healthy portion augmented. Chronic pulmonary tuberculosis is a disease of lulls and exacerbations. Some cases at times suffer mostly from insufficient breathing capacity and consequent imperfect oxygenation. The condition is associated with lack of breath, anemia, cardiac palpitation and digestive disturbances. If the healthy portion of the lungs can be made to perform more work, a positive improvement may be gained and many times in selected cases much may be accomplished. It has been claimed that the pneumatic cabinet has been useful in promoting the comfort of consumptives and I have seen marked improvement in some cases, who used it regularly for a time. The patient breathed with greater ease, could exercise more, and better nutrition was shown by gain in weight. The change was probably due to greater breathing capacity and a larger supply of air. Several years' experience has convinced the writer that the best results are obtained by the combined use of the pneumatic cabinet and the patient's own efforts. The pneumatic cabinet affords a means for supplying rarefied or compressed air. Respiration may be rendered difficult or easy and inspiration may be made easy and expiration difficult or vice versa. The patient can be compelled to breathe deeply by rarefying the air in the cabinet and thus removing a part of the atmospheric pressure from the chest-wall, and the expiratory effort may be much increased by directing the expired air into the room. Again, both the inspiratory and expiratory effort may be diminished by differentiation. The other advantages to be gained by the use of the cabinet are as follows: the patient is under control and is forced to follow directions. More complete distention of the lungs can be obtained. The amount of exertion can be measured. The patient learns more readily to breathe deeply by cabinet treatment and the vital capacity increases more rapidly, as shown by the manometer, the spirometer and measurements.

The one great disadvantage in the use of the pneumatic cabinet lies in the fact that it cannot be employed save in the physician's office. Patients are often unable to come to the consulting-room for treatment, or when able to do so the regularity of the treatment is often made impossible by the condition of the weather. The proper place to test the full value of the pneumatic cabinet in the treatment of selected cases is in an institution where the treatment may be prolonged and repeated at will.

56 Allen Street.

---

Objective Noise in the Ear.—Tomka reports the observations of an objective noise in the ear of a boy of six years, due, he thinks, to the contraction of the tensor veli muscle.—*Archiv f. Ohrenh.*, Bd. 40, p. 57.

